# Black Haw

**Published:** 1881-11-20

**Authors:** Robert Boal

**Affiliations:** Peoria, Ill.


### BLACK HAW.

BY ROBERT BOAL, M. D., PEORIA, ILL.

Among the indigenous remedies introduced to the profession, no one is entitled to greater confidence, or has received comparatively so little attention as the viburnum prunifolium, or black haw. When first brought to notice by a southern physician as a remedy to prevent miscarriage, that was supposed to be its sole remedial virtue. Experience has proved its applicability to other uterine affections. Not only is its anti-abortifacient power well attested by several observers, but in other uterine affections it is of undoubted efficacy. I have treated a few cases of threatened abortion and premature labor with the viburnum, with successful results. In my judgment it will quiet uterine action with more certainty than any other anodyne or anti-spasmodic. In a recent conversation with a medical friend, he extolled its virtues highly, and said that it seldom disappointed him in its effects. Every physician who has practiced for any length of time has been disappointed in the effect of opium in arresting threatened abortion, and many entertain the opinion, that in many cases it facilitates, rather than prevents, its occurrence. If the statements of many who have tried the viburnum are reliable, we have a remedy for a pregnant woman that is invaluable, and one that may save the lives of many children.

In dysmenorrhœa, especially in the spasmodic form which occurs in women of delicate habit and sensitive nervous organization, its action is usually prompt and beneficial. In the majority of cases of what is called functional dysmenorhœa, it will afford decided relief, if properly given, and in those cases that are due to flexions, narrowing of cervical canal, or other organic causes, its palliative effect is often well marked. The sympathetic disturbance of other organs preceding and during the menstrual flow, particularly the instability of the stomach, renders the exhibition of remedies no easy task. Many women have an idiosyncracy with regard to any preparation of opium, which renders its administration unpleasant and often useless in relieving pain in consequence of the nausea, vomiting and other unpleasant effects following its use. It is in these cases the black haw is peculiarly well adapted. Although its taste is bitter, it usually agrees with the stomach, and according to my experience is seldom rejected or produces nausea or other disagreeable results.

Its mode of action is anodyne and antispasmodic. Beyond its power of relieving pain, I have observed no other apparent effect upon the system. While the remedial power of viburnum is inestimable, it commends itself to the profession in another point of view. If it will relieve pain preceding and during menstruation, for which opium and alcoholic stimulants are ordinarily given, it will remove the danger of contracting the opium habit, or the intemperate use of intoxicating drinks, which have blighted the lives and destroyed the health and happiness of so many. If the viburnum will supply the place of these potent agents, and accomplish all, even more than they; if danger to pregnant women can be averted and the lives of children saved; if the agonizing pain can be palliated or relieved, and the formation of habits as strong as death can be prevented or avoided—then we have a boon in this agent whose value it would be hard to estimate. Whether it will accomplish *all* that is claimed for it more extended observation and experience in its use will perhaps be necessary. That it will do *much* in the affections for which it has been used, I *know* from experience. The fluid extract is the preparation I have used, giving it in half drachm to one drachm doses, repeated every one, two, three or more hours, as the exigencies of the case may require. In consequence of its bitter taste it is best combined with some of the syrups, or what is better, the aromatic or simple elixirs, in equal parts.—*Peoria Medical Monthly.*

